# Is There Still Room for Novel Viral Pathogens in Pediatric Respiratory Tract Infections?

**DOI:** 10.1371/journal.pone.0113570

**Published:** 2014-11-20

**Authors:** Blanca Taboada, Marco A. Espinoza, Pavel Isa, Fernando E. Aponte, María A. Arias-Ortiz, Jesús Monge-Martínez, Rubén Rodríguez-Vázquez, Fidel Díaz-Hernández, Fernando Zárate-Vidal, Rosa María Wong-Chew, Verónica Firo-Reyes, Carlos N. del Río-Almendárez, Jesús Gaitán-Meza, Alberto Villaseñor-Sierra, Gerardo Martínez-Aguilar, Ma. del Carmen Salas-Mier, Daniel E. Noyola, Luis F. Pérez-Gónzalez, Susana López, José I. Santos-Preciado, Carlos F. Arias

**Affiliations:** 1 Instituto de Biotecnología, Universidad Nacional Autónoma de México, Cuernavaca, Morelos, Mexico; 2 Colegio de Pediatría del Estado de Veracruz, Veracruz, Mexico; 3 Facultad de Medicina, Universidad Nacional Autónoma de México, México D.F., Mexico; 4 Hospital General de México, México D.F., Mexico; 5 Hospital Pediátirco de Coyoacán, México D.F., Mexico; 6 Nuevo Hospital Civil de Guadalajara "Dr. Juan I. Menchaca", Guadalajara, Jalisco, Mexico; 7 Centro de Investigación Biomédica de Occidente, IMSS, Guadalajara, Jalisco, Mexico; 8 Unidad de Investigación Biomédica IMSS, Durango, Durango, Mexico; 9 Universidad Autónoma de San Luis Potosí, San Luis Potosí, Mexico; 10 Hospital Central “Dr. Ignacio Morones Prieto”, San Luis Potosí, Mexico; Columbia University, United States of America

## Abstract

Viruses are the most frequent cause of respiratory disease in children. However, despite the advanced diagnostic methods currently in use, in 20 to 50% of respiratory samples a specific pathogen cannot be detected. In this work, we used a metagenomic approach and deep sequencing to examine respiratory samples from children with lower and upper respiratory tract infections that had been previously found negative for 6 bacteria and 15 respiratory viruses by PCR. Nasal washings from 25 children (out of 250) hospitalized with a diagnosis of pneumonia and nasopharyngeal swabs from 46 outpatient children (out of 526) were studied. DNA reads for at least one virus commonly associated to respiratory infections was found in 20 of 25 hospitalized patients, while reads for pathogenic respiratory bacteria were detected in the remaining 5 children. For outpatients, all the samples were pooled into 25 DNA libraries for sequencing. In this case, in 22 of the 25 sequenced libraries at least one respiratory virus was identified, while in all other, but one, pathogenic bacteria were detected. In both patient groups reads for respiratory syncytial virus, coronavirus-OC43, and rhinovirus were identified. In addition, viruses less frequently associated to respiratory infections were also found. Saffold virus was detected in outpatient but not in hospitalized children. Anellovirus, rotavirus, and astrovirus, as well as several animal and plant viruses were detected in both groups. No novel viruses were identified. Adding up the deep sequencing results to the PCR data, 79.2% of 250 hospitalized and 76.6% of 526 ambulatory patients were positive for viruses, and all other children, but one, had pathogenic respiratory bacteria identified. These results suggest that at least in the type of populations studied and with the sampling methods used the odds of finding novel, clinically relevant viruses, in pediatric respiratory infections are low.

## Introduction

Acute respiratory infections (ARIs) are the most common illnesses in humans and are associated with significant morbidity and mortality in young children in developing countries and elderly people in developed countries. In children, 156 million episodes of pneumonia are recorded annually worldwide, of which more than 95% are reported in developing countries [1], [2]. In 2008, 1.6 million children younger than 5 years died from pneumonia [3]. To try to reduce child mortality due to ARIs, is important to perform a more accurate diagnosis of the pathogens associated with those deaths in children younger than 5 years of age [1].

Introduction of PCR-based diagnostic methods has increased the ability to detect respiratory viruses, which are responsible for most ARIs in young children [4], [5], [6]. Several respiratory viruses, such as influenza, parainfluenza virus, adenovirus, respiratory syncytial virus (RSV) and coronavirus (HCoV) have been known for some time as etiological agents of lower tract respiratory infections (LRTI). More recently, with the improvement of diagnostic methods, rhinovirus (RV), which had been thought to be mostly associated with mild-to-moderate upper respiratory tract infections (URTI) was also found to be associated with severe respiratory infections [7], [8] and, in the last decade, several new respiratory viruses have been identified, such as human metapneumovirus (hMPV), HCoV-NL63 and -HKU1, human bocavirus (HBoV), parechovirus (HPeV), polyomavirus KI and WU, and enterovirus 104 and 109 [9], [10], [11], [12], [13], [14], [15]. In this regard, the fact that even with state-of-the-art diagnostic tools in most studies a virus is detected in only 50% to 80% of upper and lower ARIs [4], [5], [6], [16], [17], [18], [19] a wonder is if there are more respiratory viruses associated to ARIs than those currently known [20].

In this work, we analyzed by next generation sequencing (NGS) nasopharyngeal samples from children with LRTI and URTI that had been found negative for a panel of 21 respiratory pathogens (15 viruses and 6 bacteria) using commercial multiplex PCR methods. This study contributes to the description of the viral and bacterial populations present in nasopharyngeal samples from children with lower and upper ARIs using a metagenomic approach, which so far has been employed in limited studies [21], [22], [23], and suggests that the current diagnostic methods likely miss known respiratory pathogens, which might explain the relatively high proportion of undiagnosed cases.

## Materials and Methods

### Study populations and clinical samples

Two pediatric populations with symptomatic respiratory tract infections were included in this study. The first consisted of children with LTRI that required hospital admission due to clinical or radiological signs or symptoms of pneumonia in four different states of Mexico. Nasal washings with 1.5 ml of saline solution were collected from 250 children (male:female ratio, 1.43; age range, 1–76 months) between March 2010 and April 2011. The second population was composed of patients with symptomatic URTI that attended the private consult in five different cities of the state of Veracruz, Mexico. Nasopharyngeal swabs (rayon-tipped, BD BBL) were collected from 526 children (male:female ratio, 1.27; age range, 0–191 months) from September 2011 to April 2012. All samples were placed in vials containing viral transport medium (1∶1 in the case of nashal washings; Microtest M4-RT, Remel) and sent frozen in blue ice either to the Institute of Biotechnology in Cuernavaca (URTI samples) or to the School of Medicine in Mexico City (LRTI samples) and stored at −70°C until analyzed. All children were previously healthy, not diagnosed with tuberculosis or signs of malnutrition, and not immunocompromised. Administration of antibiotics before hospital admission was not registered; in outpatients no antibiotics were administered before sample collection. The children included in the study were those that arrived consecutively at the collection places during the study period, with no further selection. The study (project 186) was approved by the institutional review boards of the School of Medicine and the Institute of Biotechnology of the National University of Mexico and from the institutional review board and ethics committee of each participant hospital. Written informed consent was obtained from each parent or guardian prior to enrollment.

### Pathogen detection

The respiratory specimens from hospitalized and outpatient children were previously screened for viruses using the xTAG Bioplex respiratory Viral Panel (Abbott, Rungis, France) (JI Santos et al., in preparation) and the Seeplex RV15 ACE detection kit (Seegene, Seoul, Korea) (Wong-Chew et al., in preparation), respectively. The virus-negative samples from both groups of patients were screened in this work by a multiplex PCR (Seeplex Pneumobacter ACE detection kit, Seegene, Seoul, Korea) for the presence of six bacteria commonly associated to respiratory infections: *Streptococcus pneumoniae, Haemophilus influenzae, Chlamydophila pneumoniae, Legionella pneumophila, Bordetella pertussis*, and *Mycoplasma pneumoniae*.

### Nucleic acid extraction, amplification and barcode labeling

Genetic material from clinical samples was extracted with the PureLink Viral RNA/DNA kit according to the manufacturer's instructions (Invitrogen, Waltham, MA). Before extraction, samples (200 µl) were treated with Turbo DNAse (Ambion, Waltham, MA) and RNAse (Sigma, St. Louis, MO) for 30 min at 37°C and immediately chilled on ice. Nucleic acids were eluted in nuclease-free water, aliquoted, quantified in NanoDrop ND-1000 (NanoDrop Technologies, Waltham, MA), and stored at −70°C until further use. Sample random primer-amplification of nucleic acids was performed essentially as described previously [24]. Briefly, reverse transcription was done using SuperScript III Reverse Transcriptase (Invitrogen, Waltham, MA) and primer-A (5'-GTTTCCCAGTAGGTCTCN_9_-3'). Complementary DNA (cDNA) strand was generated by two rounds of synthesis with Sequenase 2.0 (USB, USA). The cDNA obtained was then amplified with KlenTaq polymerase (Sigma, St. Louis, MO) using the primer-B (5'-GTTTCCCAGTAGGTCTC-3') and 20 cycles of the following program: 30 sec at 94°C, 1 min at 50°C, 1 min at 72°C. After cleaning the PCR products with the DNA Clean & Concentrator-5 kit (Zymo Research, Irvine, CA), DNA was digested with the GsuI restriction enzyme (Fermentas Waltham, MA) for 2 h at 30°C to remove sequences corresponding to PCR primers. After digestion, samples were purified again and used as starting material to prepare 300 bp-sized libraries using Illumina's Genomic DNA sample Prep Kit with multiplex primers as suggested by the manufacturer (Illumina, San Diego, CA). Libraries were loaded in a flow cell (4 or 5 libraries per lane) and sequencing was performed by 72 cycles of nucleotide extension followed by acquisition of multiplex code in a Genome Analyzer IIx. The datasets generated by the GAIIx were deposited in the European Nucleotide Archive, with study accession numbers PRJEB7390 and PRJEB7391 for URTI and LRTI samples respectively.

### Deep sequencing and sequence analysis

Image analysis and base calling were performed with the Illumina GAPipeline program (version 1.3.0) using standard parameters. To separate the samples, the pooled data from each lane were binned by barcode. In-house scripts were developed for the sequence analysis, including the following steps:

*i) Preprocessing*. For each read, the adapter and 5' and 3' bases with no-call sites (N residues) and low-quality (Phred-like scores <20) were trimmed. Then, low complexity reads and less than 35 bases long were removed. Finally, identical reads were collapsed into a single representative sequence to optimize analysis time. Only reads passing the preprocessing step were considered valid.

*ii) Removal of host sequences*. The program SMALT (Wellcome Trust Sanger Institute, 2012) was used to align the reads against mitochondrial, human genome, and bacterial ribosomal RNA to remove them, using 90% coverage and 90% identity.

*iii) Taxonomic identification*. To minimize CPU time, valid reads were aligned to bacteria, fungi and viruses nt NCBI databases, using SMALT with 70% coverage and identity. Then, the reads that mapped were aligned with standalone BLASTn against the databases described above, using an E-value of 1e–03. To avoid misclassification, the first 100 hits were obtained for each sequence. Reads that did not map were considered as unidentified.

*iv) Taxonomic classification*. To assign reads to the most appropriate taxonomic level the software MEGAN 4.70.4 was used, which assigns a read to the lowest common taxonomic ancestor of the organisms corresponding to the set of significant hits.

*v) Assembly*. Reads assigned to the same virus family level were subsequently used for *de novo* assembly with Velvet 1.1.04 to increase the accuracy of classification. Each assembly contig was aligned against BLASTn database.

*vi) Detection of novel viruses*. All unidentified sequences unaligned using SMALT nucleotide alignment were assembled *de novo* with Metavelvet modified by us to improve the assembly efficiency. First, we conducted exploratory assemblies of the reads using multiple hash lengths (k = 17–35). Then, additional assembly of all unused reads from the exploratory assemblies was done (k = 21). Finally, we assembled all contigs obtained from all exploratory assemblies and the unused reads assembly by using the program VelvetOptimiser. From this final assembly, contigs that were greater than 180 nt were directly compared with NCBI nr (non- redundant protein) database using BLASTx with an E-value of 100 in an attempt to identify novel viruses.

### Phylogenetic tree inference

Metagenomic contigs from specific viruses that were at least 150 nt-long, were phylogenetically characterized. The analysis required a different approach compared to full-length genomes due to the fact that metagenomics sequences are fragmentary and not completely overlapping. Therefore, for each virus, a database of complete genomes was first created using all sequences available in GenBank until January 2014. Then, a reference alignment was done with sequences of this database by using MUSCLE method. Next, we combined metagenomics contigs into a single large alignment by using the software MAFFT with the option align fragment sequences to reference alignment. Finally, maximum likelihood trees were generated with 1000 repetitions bootstrap using the MEGA program.

## Results

### Pathogen detection

In previous studies we screened by RT-PCR the presence of 15 respiratory viruses in nasal washings from 250 hospitalized children with clinical diagnosis suggestive of viral pneumonia and in 526 nasopharyngeal samples from pediatric children with URTI (see Materials and Methods). Table 1 shows the frequency of the different viruses found in both types of samples. Among the viruses detected, considering both single and multiple infections, RSV-A and rhinovirus showed the highest frequency in both LRTI and URTI. At least one virus was detected in 71.2% (178/250) of LRTI (Santos et al., manuscript in preparation) and 71.5% (376/526) of URTI (Wong-Chew et al., manuscript in preparation). In 40 of the 250 LRTI samples (16%) a viral coinfection was found. Thirty-four of these samples had a dual infection, with the combination of RSV-A/RV and RSV-A/AdV being the more frequent, while 6 children had triple virus infections. In the case of URTI, 73 of the 526 samples (13.9%) showed a viral coinfection. Sixty-three of these samples had a dual infection, with the combination of AdV/EV and RV/CoV 229/N63 being the more frequent. Eight children had triple virus infections, and two were infected simultaneously with four viruses.

**Table 1:** Frequency of viral pathogens in children with URTI and LRTI.

Virus	URTI (%) a	LRTI (%)
Respiratory syncytial virus-A	96 (18.3)	77 (30.8)
Rhinovirus	92 (17.5)	62 (24.8)
Influenza virus A	48 (9.1)	4 (1.6)
Adenovirus	38 (7.2)	14 (5.6)
Enterovirus	31 (5.9)	2 (0.8)
Metapneumovirus	28 (5.3)	19 (7.6)
Coronavirus 229E/NL63	28 (5.3)	2 (0.8)
Coronavirus OC43	18 (3.4)	4 (1.6)
Parainfluenza virus 3	18 (3.4)	27 (10.8)
Parainfluenza virus 1	15 (2.9)	8 (3.2)
Bocavirus	13 (2.5)	8 (3.2)
Parainfluenza virus 4	13 (2.5)	2 (0.8)
Parainfluenza virus 2	9 (1.7)	7 (2.8)
Influenza virus B	7 (1.3)	4 (1.6)
Respiratory syncytial virus-B	7 (1.3)	2 (0.8)

a The number of viruses include those present in single and mixed infections. The percentage refers to the total number of viruses detected.

The virus-negative samples were screened by a multiplex PCR for the presence of six bacteria commonly associated to respiratory infections. In 64.7% (46/71, LRTI) and 68.7% (103/150, URTI) of the virus-negative samples at least one bacterial pathogen was found. The most frequent bacteria detected in children in both types of populations were *S. pneumoniae* (36 LRTI, 88 URTI) and *H. influenzae* (24 LRTI, 47 URTI); in a few cases *C. pneumoniae* (9 URTI) and *M. pneumoniae* (2 LRTI, 2 URTI) were also detected. In 37 children with URTI two different bacteria were found, and in 3 children 3 bacteria were detected. In the case of LRTI, 8 children had a mixed infection. It is important to have in mind that bacterial colonization, frequently at lower bacterial colony counts, may be detected by very sensitive laboratory tests, and even more frequently than viruses, these bacteria may not be associated with acute disease.

After screening for common respiratory viruses and bacteria, 90% of children with LRTI and 91.3% with URTI had at least one pathogen identified. The remaining 25 (10%) hospitalized and 46 (8.7%) outpatient children remained negative for all the tested pathogens and were then characterized by next-generation sequencing (NGS).

### Next-generation sequencing of negative samples

To search for either known or novel respiratory pathogens in the double-negative (virus and bacteria) samples, the nucleic acids in these samples were isolated, amplified by PCR, and sequenced using the Illumina platform, as described in Materials and Methods. The 25 samples from children with LRTI were sequenced individually (listed in Table 2). In the case of the URTI samples, 9 were sequenced individually, while the amount of DNA isolated from the other 37 samples was too low to be analyzed independently, thus, they were used to prepare 16 pools for sequencing: 13 pools of two samples, 1 pool of three samples, and 2 pools of four samples (Table 3).

**Table 2:** DNA reads obtained after NGS sequencing of LRTI samples.

Sample	No. of reads	aNo. of valid reads (%)
11	7,336,101	6,243,183 (85.8)
17	13,118,032	10,877,350 (83.5)
24	8,522,571	580,599 (7.0)
28	4,210,763	3,400,829 (81.3)
47	9,051,977	1,474,628(16.3)
64	10,626,262	1,125,214 (10.7)
66	11,937,236	713,915 (6.1)
67	10,779,916	696,529 (6.6)
86	16,500,963	1,806,051 (11.1)
111	13,312,546	941,951 (7.3)
124	17,270,372	1,943,449 (11.4)
125	10,053,798	627,927 (6.4)
147	6,229,459	464,760 (7.5)
151	14,208,710	1,248,513(8.9)
206	2,881,815	137,274 (9.1)
210	16,684,541	9,969,067 (60.2)
211	10,784,070	1,099,819 (10.4)
213	9,137,653	775,688 (8.6)
214	11,503,832	4,321,038 (37.8)
225	18,787,796	1,534,587 (8.4)
227	19,294,597	1,483,230 (7.9)
233	14,731,213	3,628,367 (24.9)
236	13,712,286	974,744 (7.2)
237	17,095,111	2,780,835 (16.8)
238	12,373,402	3,277m334 (26.7)

a Valid DNA reads after discarding those that did not pass the quality filter, and removing repeated reads (see Methods).

**Table 3:** DNA reads obtained after NGS sequencing of URTI samples.

aIndividual and pooled samples	No. of reads	bNo. of valid reads (%)
C06, C55, T78, V24	4,323,309	1,707,160 (39.5)
C16, C61	2,113,065	1,134,387 (53.7)
C27, C01	2,951,784	1,474,233 (49.9)
C29, M40, T41, V39	3,325,315	1,235,867 (37.2)
C41, T50	3,765,099	1,708,531 (45.4)
C46, P54	4,289,979	2,278,068 (53.1)
M23, M44	3,607,011	1,982,487 (55.0)
M28	7,294,104	3,292,828(45.1)
P06, P150	5,010,364	2,387,550 (47.7)
P108	4,609,150	1,021,900 (22.2)
P147, P191	3,454,018	1,540,283 (44.6)
P149, P153	4,381,981	2,534,733 (57.8)
P151, P181	4,638,675	2,207,800 (47.6)
P173	2,974,252	613,487 (20.6)
P176, P186, P213	4,210,620	2,176,224 (51.7)
P183	1,654,081	272,720 (16.5)
P19, P88	8,727,411	5,067,242 (58.1)
P69	3,072,261	631,373 (20.6)
T33, T39	4,863,936	3,308,371 (68.0)
T36	1,228,687	446,932 (36.4)
T38	3,309,781	1,420,555 (42.9)
T43, T44	3,183,181	2,325,025 (73.0)
T65	78,467	30,443 (38.8)
V26	3,469,556	1,496,608 (43.1)
P131, V61	3,939,137	2,288,528 (58.1)

a The samples that were pooled for sequencing are indicated.

b Valid DNA reads after discarding those that did not pass the quality filter, and removing repeated reads (see Methods).

**Figure 1:**
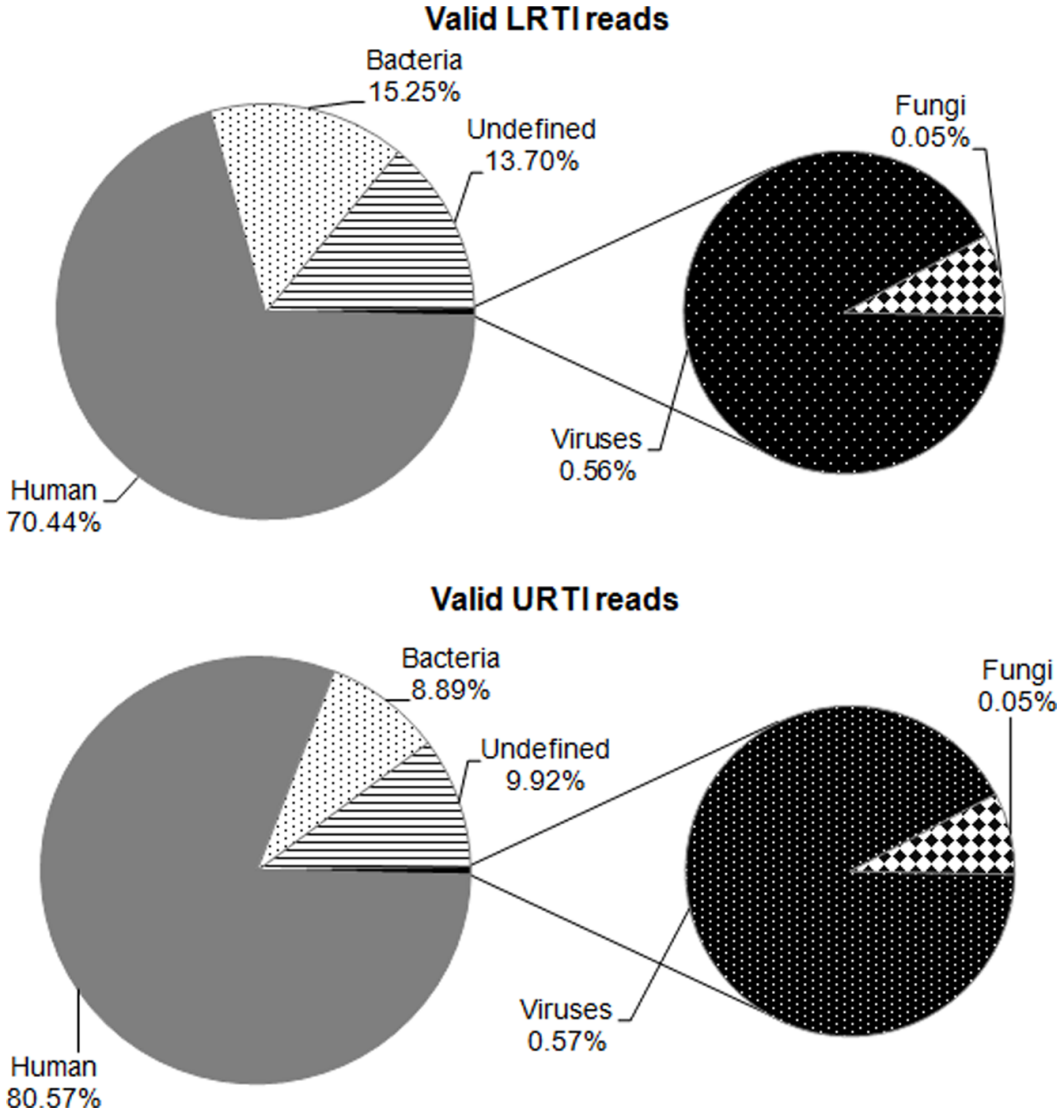
Taxonomic classification of the generated DNA sequencing reads. Valid DNA reads obtained by NGS of LRTI and URTI clinical samples were split into human, bacterial, fungal, and viral origin. Those reads not present in the four previous categories were classified as "undefined". Average values for all LRTI and URTI samples are shown.

The total number of DNA reads and the valid unique reads obtained from each sample after passing the quality controls are shown in Tables 2 and 3. The valid reads were analyzed for the presence of sequences from human, bacterial, fungal, or viral origin. As expected, the most abundant reads were from human origin, representing 70% and 80% of LRTI and URTI patients, respectively (Fig. 1). Bacterial sequences made up the second largest data set, representing 15.2% of the sequence reads in LRTI and 8.5% in URTI. Viral sequences represented 0.56% and 0.57% of valid reads in LRTI and URTI, respectively, and only 0.05% of reads corresponded to fungi (Fig. 1). Finally, approximately 13% and 10% of the sequences in both LRTI and URTI could not be classified since no homolog was found (E-value 1e–03) or there were contradicting database hits. This category is referred to as ‘undefined’ in Figure 1. Of interest, despite the fact that the samples from LRTI and URTI were collected by different methods (nasal washings vs. swabs), and from children with different clinical syndromes and varying severity of respiratory disease, the proportion of sequences from different origins was very similar.

The undefined sequence reads from all samples were assembled, and contigs ≥180 nt were compared with non-redundant protein database of GenBank (E-value 100) to find sequences that could be distantly related to known viral sequences and could thus represent novel viruses. Indeed, short sequences are less likely than long sequences to retrieve statistically significant similarities in Blast searches, and sequence assembly into longer contigs is helpful to overcome this difficulty. As result of this, all filtered contigs aligned either to bacterial or human proteins during BLASTx runs. An analysis revealed that the contigs that map to bacteria showed only 60–80% nucleotide identity to their best-matching reference, indicating that they most likely represent novel species within their corresponding genera and thus could not be classified during alignments with BLASTn. Nonetheless, the vast majority of reads (50% to 90%) were not assembled into contigs. The unassembled reads were low complexity sequences or library artifacts as adapter chimeras, suggesting that it is unlikely that they correspond to novel viruses. A remaining small amount of sequences could not be assembled due to non-uniform read depth because of a non-uniform species abundance distribution.

### Viruses detected by NGS in double-negative samples

DNA sequence reads from at least one virus commonly associated to respiratory infections was found in 20 out of the 25 double-negative samples of LRTI patients (Table 4): 5 samples were positive for RSV reads, 11 samples for HCoV-OC-43, and 9 for RV. In addition, 5 samples contained HBoV and in 12 samples anelloviruses (torque teno -TTV-, torque teno mini -TTMV-, or torque teno midi viruses -TTMDV) were also detected; rotavirus, papillomavirus, and herpesvirus sequences were identified once in the samples, and reads from several viruses from both animal (bat picornavirus, bovine viral diarrheal virus, bovine kobovirus) and plant origin (potato virus Y, pepper mild mottle virus), as well as various bacteriophages were also found (Table 4). Regarding bacteria, DNA sequence reads from *S. pneumoniae* were the most frequent, being present in all but one of the 25 samples sequenced, and *M. catarrhalis, L. pneumoniae*, and *H. influenzae* were less frequently found. DNA reads from other bacteria less commonly associated with respiratory infections were also detected (Table 4). Some of the samples had sequence reads corresponding to up to 8 different viruses or 15 different bacteria. Of interest, including the NGS results, 79.2% (198/250) of the samples had a respiratory virus detected, and in the remaining 52 samples at least one bacteria was found, such that all 250 samples from children with LRTI had a respiratory pathogen identified.

**Table 4:** Pathogens identified by NGS in children with lower respiratory tract infections.

	Samples																								
Pathogen detected	47	86	124	111	28	206	210	211	213	214	225	227	233	236	237	238	17	24	11	125	147	151	64	66	67
**VIRUSES**																									
**Respiratory syncytial virus**	3^a^	372	-	-	-	-	-	-	-	-	-	-	-	-	-	-	12	-	-	-	-	7	-	318	-
**Coronavirus OC43**	-	-	-	-	5	-	45	4	3	1222	-	-	-	-	-	239049	1672	2	392	600	-	-	-	-	3
**Rhinovirus A**	5	-	66	-	2	-	-	16009	-	-	-	3	206	-	16	-	-	-	-	-	-	3	-	-	-
**Rhinovirus C**	-	-	121	-	-	-	-	63	-	-	3	-	38475	-	39	-	-	-	-	-	-	-	-	-	-
**Human bocavirus**	-	-	-	-	-	-	162	-	-	87209	6	-	-	-	-	322	483	-	46	-	-	-	-	-	-
**Torque teno virus (TTV)**	-	-	-	-	-	-	5	3	616	7	-	1007	125	-	-	15	7	32	-	-	2	-	9	160	-
**Torque teno mini virus (TTMV)**	-	-	-	-	-	-	46	-	6	-	-	-	-	-	-	3	-	7	-	-	-	-	2	54	-
**Torque teno midi virus (TTMDV)**	-	-	-	-	-	-	-	-	-	-	-	-	9	-	-	-	-	-	-	-	-	-	-	6	-
**Human herpes virus**	-	-	-	-	-	-	-	-	-	-	-	-	-	-	-	-	-	-	2	-	-	-	-	-	-
**Human papilloma virus**	-	-	-	-	-	-	-	-	-	-	-	31	-	-	-	-	-	-	-	-	-	-	-	-	-
**Rotavirus**	-	-	-	-	-	-	-	3	-	-	-	-	-	-	-	-	-	-	-	-	-	-	-	-	-
**Bat picornavirus**	-	-	-	-	-	-	-	4	-	-	-	-	-	-	-	-	-	-	-	-	-	-	-	-	-
**Bovine kobuvirus**	-	-	-	-	-	-	6	130	-	-	-	-	-	-	-	-	-	-	-	-	-	-	-	-	-
**Bovine viral diarrhea virus**	-	-	-	-	-	-	5	20	-	-	-	-	-	-	-	-	-	-	-	-	-	-	-	-	-
**Pepper mottle virus**	-	-	-	-	-	-	-	-	-	-	-	-	8	-	-	-	-	-	-	-	-	-	-	-	-
**Potato virus Y**	-	-	-	7	-	-	-	-	-	-	-	-	-	-	-	-	-	-	-	-	-	-	-	-	-
**Phages**	-	236	294	955	-	-	33	109	83	9	82	394	32	117	25	17	-	335	2	2384	569	383	67	1201	1345
																									
**BACTERIA**																									
**Streptococcus pneumoniae**	3	10	14	7	-	57	4	34	25	37	3	22	25	6	94	418	4	25	23	27	420	48	186	104	3,964
**Haemophilus influenzae**	4	130	41	210	-	-	-	5	35	17	-	13	-	-	-	-	-	41	-	-	-	-	105	-	-
**Moraxella catarrhalis**	110	11,845	380	-	-	-	-	23	2,213	12	1,237	3	1,909	5	70	-	58	6	-	10	-	3	-	6	13
**Legionella pneumophila**	-	5	5	-	-	-	3	21	-	-	3	24	-	4	-	-	-	-	-	-	-	118	-	-	-
**Klebsiella pneumoniae**	-	-	-	37	-	5	-	5	6	-	2	-	-	4	-	-	-	-	3	-	-	-	-	-	-
**Staphylococcus aureus**	-	-	14	31	-	-	64	12	85	2	248	-	17	322	5	-	-	52	-	43	4	-	31	-	22
**Rahnella**	-	14	-	2,239	-	-	-	-	5	-	3	3	-	-	-	-	-	906	-	8	-	-	-	15	4
**Burkholderia cepacia**	3	228	242	1,753	-	133	29	475	236	22	420	1,135	98	148	16	-	-	325	-	25	163	15	255	1,168	106
**Acinetobacter baumannii**	67	1,419	1,092	4,622	-	685	152	1,491	1,660	57	1,252	1,793	579	46	37	54	-	2,248	-	2,210	180	350	347	783	2,425
**Pseudomonas aeruginosa**	14	398	222	1,044	-	531	61	282	430	9	719	1,525	123	914	26	10	1	-	-	965	63	199	307	1,175	369
**Mycobacterium tuberculosis**	-	7	-	11	-	-	-	-	3	-	7	-	-	-	-	-	-	-	-	-	-	-	-	-	-
**Actinomycetales**	15,563	25	52	316	-	38	-	61	161	16	77	151	17	19	32	-	-	-	-	33	-	10	15	-	-
**Burkholderia gladioli**	22	3,716	3,897	36,947	-	174	320	7,756	2,264	439	10,157	13,733	1,661	2,473	235	127	5	107	-	645	355	355	3,143	460	500
**Malassezia globosa**	35	154	270	-	447	-	1,554	48	-	785	208	146	848	-	214	620	1,915	-	1,062	21	102	392	141	142	87
**Pseudomonas mendocina**	-	98	67	359	-	154	12	112	86	-	291	430	27	90	10	-	-	63	-	623	19	74	93	50	75
**Staphylococcus epidermidis**	-	88	-	2681	-	-	5,007	-	1,341	-	-	-	-	-	88	-	-	-	-	15	-	210	-	40	111
**Leifsonia xyli**	2,494	-	-	-	-	-	-	-	-	-	-	-	-	-	-	-	-	-	-	-	-	-	-	-	-
**Acidovorax**	69	1,280	885	6,704	-	808	222	1,325	2,042	106	3,814	5,079	543	551	59	40	17	7,074	-	7,704	2,057	995	771	5,345	5,061

a Number of valids DNA reads in the sample por the corresponding pathogen.

DNA reads from one to five typical respiratory viruses were detected in 22 of the 25 sequenced double-negative individual and/or pooled samples from children with URTI (Table 5): The virus most frequently detected was RV, which was found in 19 of the pooled and/or individual samples; some of the samples had more than one type of virus, such that we found sequence reads from 4 RV subtype A, 4 subtype B, and 19 subtype C. One sample was positive for RSV, 3 for HCoV-OC43, 3 for human enterovirus A71, and 3 samples had HBoV. Of interest, 5 of the samples had DNA reads from Saffold virus, a virus recently described to be associated to respiratory infections. Also, among these samples we identified 3 containing herpesvirus, 5 papillomavirus, 2 human astrovirus, 4 rotavirus, and 10 anelloviruses (TTV, TTMV, TTMDV). Similar to what was found in LRTI, in children with URTI DNA reads of viruses from animal (white spot syndrome and bat picornavirus) and plant origin (Okra mosaic virus, capsicum chlorosis virus, cucumber mosaic virus, pepper mild mottle virus, and tomato mosaic virus) were also detected. In the majority of samples bacteriophages were found. After NGS, all pooled samples had DNA sequences from at least one respiratory virus, while three individual (not pooled) samples remained negative for common respiratory viruses (Table 5). In two of these samples (P183 and T38) *M. catarrhalis* was detected and *S. pneumoniae* was additionally present in one of them. The third sample (T65) remained negative for both viruses (including phages) and bacteria. Some of the samples from URTI had sequence reads corresponding to up to 11 different viruses or 18 different bacteria. Considering the NGS results, and assuming that in the pooled samples the identified viruses were each present in a different sample, 76.6% (403/526) of the children had a common respiratory virus detected, while in all but one of the other 129 children a respiratory bacteria was identified.

**Table 5:** Pathogens identified by NGS in children with upper respiratory tract infections.

	Samples^a^																								
Pathogen detected	C06, C55, T78, V24	C16, C61	C27, C01	C29, M40, M41, V39	C41, T50	C46, P54	M23, M44	M28	P06, P150	P108	P131, V61	P147, P191	P14,9 P153	P151, P181	P173	P176, P186, P213	P183	P19, P88	T69	T33, T39	T36	T38	T43, T44	T65	V26
**VIRUSES**																									
**Respiratory syncytial virus**	-	-	-	-	-	-	-	-	-	-	-	-	-	-	-	-	-	-	-	3	-	-	-	-	-
**Coronavirus OC43**	-	-	-	-	-	-	-	-	-	-	-	3	24	-	38	-	-	-	-	-	-	-	-	-	-
**Rhinovirus A**	-	-	-	-	-	-	-	-	-	-	49	59	-	1,052	-	-	-	-	2	-	-	-	-	-	-
**Rhinovirus B**	-	-	-	-	-	-	-	-	14	-	3,995	12	-	8	-	-	-	-	-	-	-	-	-	-	-
**Rhinovirus C**	8418^b^	15	59	3,667	115,617	44	65,889	2	11,233	-	702	46	55	54	-	66	-	11,026	-	1,395	41	-	14,561	-	207
**Human enterovirus A**	-	-	-	-	15	-	-	-	15,236	-	78	-	-	-	-	-	-	-	-	-	-	-	-	-	-
**Human bocavirus**	-	-	-	-	-	-	-	-	-	12	8	-	-	-	-	-	-	-	-	-	-	-	-	-	98
**Saffold virus**	182	-	-	183	-	-	-	-	-	-	10	-	2,672	-	-	9,891	-	-	-	-	-	-	-	-	-
**Torque teno virus (TTV)**	-	-	9	-	7	187	-	-	12	-	212	14	-	7	-	-	-	-	-	3	-	-	-	-	-
**Torque teno mini virus (TTMV)**	-	-	-	3	5	100	-	-	22	-	3,408	9	-	17	-	-	-	-	-	-	-	-	-	-	-
**Torque teno midi virus (TTMDV)**	3	-	-	8	12	11	-	-	-	-	2,711	5	-	7	-	-	-	11	-	-	-	-	-	-	-
**Human herpes virus**	-	-	-	-	-	3	-	-	-	36	-	-	-	-	-	-	-	53	-	-	-	-	-	-	-
**Human papilloma virus**	-	-	3	-	-	-	6	-	-	-	3	-	-	118	-	-	-	5	-	-	-	-	-	-	-
**Rotavirus**	-	-	-	3	-	27	-	-	-	-	-	-	-	-	-	3	-	-	-	4	-	-	-	-	-
**Human astrovirus**	6	-	-	-	3	-	-	-	-	-	-	-	-	-	-	-	-	-	-	-	-	-	-	-	-
**Bat picornavirus**	-	-	-	-	-	-	-	-	-	-	4	-	-	-	-	-	-	-	-	-	-	-	7	-	-
**Capsicum chlorosis virus**	-	-	-	-	-	-	-	-	-	-	-	-	-	-	-	2	-	-	-	-	-	-	-	-	-
**Cucumber mosaic virus**	12	18	-	-	-	-	6	-	-	-	-	-	-	-	-	-	-	-	-	-	-	-	-	-	-
**Okra mosaic virus**	-	-	-	-	-	-	-	-	-	-	-	-	-	-	-	-	-	2	-	-	-	-	-	-	-
**Pepper mottle virus**	-	-	2	-	-	-	-	-	-	-	-	-	-	-	-	-	-	-	-	-	-	-	-	-	-
**Tomato mosaic virus**	-	16	12	-	-	4	-	-	3	-	-	4	-	-	-	-	-	8	-	-	-	-	-	-	-
**White spot syndrome virus**	-	-	-	-	-	-	-	-	-	-	-	-	-	-	-	31	-	-	-	-	-	-	-	-	-
**Phages**	10	28	31	54	46	102	60	6	12	11	16	327	36	139	10	568	6	21	8	43	4	-	8	-	6
																									
**BACTERIA**																									
***Streptococcus pneumoniae***	13	80	103	14	3	180	12	51	42	3	15	21	51	83	-	20	-	274	9	-	4	-	-	-	-
***Haemophilus influenzae***	15	-	4	4	3	47	15	-	4	-	-	173	10	41	-	288	47	6	-	-	-	-	-	-	-
***Moraxella catarrhalis***	235	8	15	4	34	172	22	6,192	26	10	446	-	11	6	-	6	-	142	-	76	11	8	-	-	-
***Legionella pneumophila***	8	22	-	14	-	-	22	4	-	-	15	-	-	-	-	-	-	-	-	-	-	-	-	-	-
***Klebsiella pneumoniae***	-	9	-	3	-	-	-	-	-	-	-	-	-	-	-	-	-	3	-	-	-	-	-	-	-
***Staphylococcus aureus***	9	13	4	24	16	-	36	-	-	-	-	-	20	10	-	12	-	21	-	5	-	-	288	-	-
***Rahnella***	5	-	-	-	-	-	-	-	-	-	-	-	15	-	-	-	-	-	-	-	-	-	-	-	-
***Burkholderia cepacia***	65	46	16	9	7	-	-	14	-	-	-	-	4	-	-	-	-	-	-	-	-	-	-	-	-
***Acinetobacter baumannii***	1,783	1,754	825	1,301	778	2,390	5,204	402	202	-	654	2,107	208	1,544	-	1,347	17	556	-	179	-	-	179	-	50
***Pseudomonas aeruginosa***	81	37	32	134	16	115	97	29	131	-	61	167	163	211	-	196	-	274	17	16	-	-	7	-	-
***Mycobacterium tuberculosis***	6	-	4	-	-	-	-	-	-	-	-	-	-	-	-	-	-	-	-	-	-	-	-	-	-
**Actinomycetales**	1,017	410	2,283	1,141	7,369	575	692	357	1,600	64	482	1,049	1,106	779	13	753	122	13,679	201	2,159	13	-	341	-	117
***Burkholderia gladioli***	-	4	-	9	-	-	-	-	-	-	-	-	-	-	-	-	-	-	-	-	-	-	-	-	-
***Malassezia globosa***	21	10	87	17	145	103	34	267	-	43	578	12	111	48	123	102	-	136	31	77	11	75	57	-	42
***Pseudomonas mendocina***	124	106	53	242	56	109	160	46	146	-	73	201	137	205	-	214	-	370	50	17	-	-	3	-	-
***Rhodotorula glutinis***	265	396	74	202	134	69	510	11	-	-	-	-	10	-	-	8	-	19	-	7	-	-	-	-	-
***Staphylococcus epidermidis***	27	110	62	19	149	27	57	9	52	-	18	-	50	205	-	62	-	-	29	6	-	-	3	-	-
***Lysinibacillus sphaericus***	115	301	539	29	84	117	568	234	-	-	-	-	-	-	-	-	-	18	-	-	-	-	-	-	-
***Acidovorax***	508	190	117	356	76	80	315	124	30	-	163	136	-	189	-	207	-	58	-	31	-	-	9	-	12
***Fervidobacterium nodosum***	278	677	519	345	320	999	1,152	164	349	-	270	681	1,487	1,094	-	590	-	41	93	208	-	-	121	-	-

a When more than one sample were pooled for sequencing, the code for the various samples is mentioned.

b Number of valids DNA reads in the sample por the corresponding pathogen.

### Genome assembly and phylogenetic analyses

To estimate the sequence coverage of the NGS-identified viruses, the sequence reads were assembled *de novo*, and all contigs were used to estimate the extent of the virus genome coverage. A significant coverage was obtained for several of the detected viruses. In patients with LRTI, the genome of 14 viruses was assembled with coverage higher than 20% (Table 6). As indication of the sensitivity of NGS and of the relative abundance of some viruses not detected by the conventional PCR, we could assemble more than 95% of the genome of two RV strains, 98% of one HBoV, and 99.8% of one HCoV-OC43 strain. In the case of children with URTI, at least 50% of the genome was covered for 15 viruses, including RV, HEV, Saffold virus, and TTV (Table 7), with 8 of them having a genome coverage of more than 90%. For the DNA reads of the animal viruses identified in both types of children populations the coverage ranged between 0.57 and 4.9%, and for plant viruses between 0.13 and 7.15% in the case of tomato mosaic virus (Tables 5A and B).

**Table 6:** Genome coverage for viruses present in LRTI samples.

Sample	Virus^a^	No. of Contigs	No. of reads incorporated into contigs	Genome size	Genome Coverage (%)
86	RSV	22	213	15,191	6.75
124	RV-A	6	47	7,129	7.43
	RV-C	10	96	7,107	24.34
111	PVY	1	7	9,704	1.75
210	HBoV	16	74	5,299	33.97
	HCoV	3	10	30,578	5.89
	TTMV	2	14	2,912	8.24
	BKV	1	4	8,374	1.07
	BVDV	1	3	12,230	0.57
211	RV-A	1	13,418	7,129	94.60
	RV-C	11	47	7,107	17.03
	BatPV	1	3	7,753	0.97
	BKV	5	83	8,374	4.90
	BVDV	1	9	12,230	0.61
	RV	1	3	17,360	0.40
213	TTV	12	407	3,725	32.48
214	HBoV	4	53,548	5,299	97.62
	HCoV	80	848	30,578	39.96
	TTMV	1	3	2,912	2.40
225	HBoV	1	3	5,299	1.51
	RV-C	1	2	3,725	2.01
227	TTV	8	777	3,725	34.90
	HPV	1	12	7,466	0.94
233	RV-A	16	127	7,129	26.65
	RV-C	2	30712	7,107	97.88
	TTV	16	75	3,725	52.35
237	RV-A	1	3	7,129	0.98
	RV-C	6	35	7,107	7.46
238	HBoV	22	202	5,299	38.69
	HCoV	5	85766	30,578	99.81
	TTV	1	4	3,725	2.01
17	HBoV	32	279	5,299	61.80
	HCoV	112	1,021	30,578	20.52
	RSV	1	3	15,191	0.49
24	TTV	12	1	3,725	1.88
11	HBoV	6	45	5,299	12.08
	HCoV	15	291	30,578	5.72
125	HCoV	32	382	30,578	11.28
151	RSV	1	4	15,191	0.46
66	RSV	24	274	15,191	15.40
	TTV	3	107	3,725	9.93
	TTMV	2	23	2,912	4.46

a RSV, respiratory syncytial virus; RV-A, rhinovirus species A, RV-C; rhinovirus species C; HBoV, human bocavirus, HCoV, human coronavirus OC43; TTV, torque teno virus; TTMV, torque teno mini virus; BKV, bovine kobuvirus; BVDV, bovine viral diarrhea virus; BatPV, bat picornavirus; HPV, human papillomavirus; PVY, potato virus Y.

**Table 7:** Genome coverage for viruses present in URTI samples.

Sample	Virus^a^	No. of Contigs	No. of reads incorporated into contigs	Genome size	Genome Coverage (%)
C27, C01	ToMV	2	12	6,383	1.88
	TTMDV	2	5	3256	4.45
	HPV	1	2	7466	0.94
	RV-C	9	45	7107	11.3
C41, T50	TTMV	2	5	2912	3.78
	TTMDV	2	10	3256	3.22
	TTV	1	3	3725	2.28
	RV-C	1	92130	7107	95.40
C16, C61	CMV	4	10	3356	7.15
	ToMV	3	15	6386	2.98
	RV-C	2	10	7107	2.74
M23, M44	CMV	1	3	3356	5.36
	RV-C	1	39840	7107	97.20
P108	HHV	5	12	116114	0.28
	HBoV	2	5	5299	2.64
P06, P150	HEV-A	2	12352	7,413	97.88
	TTMV	1	6	2912	4.12
	RV-C	4	8798	7107	92.74
P149, P153	HCoV	3	11	30,578	0.78
	SAFV	5	1909	8,115	83.03
	RV-C	4	26	7107	4.50
P173	HCoV	3	9	30,578	0.69
P151, P181	RV-A	7	943	7,129	50.78
	RV-B	1	4	7,215	0.97
	HPV	9	45	7466	12.46
	TTMV	2	7	2912	4.98
	TTV	1	3	3725	0.87
	RV-C	5	17	7107	6.33
P147, P191	RV-A	3	12	7,129	4.35
	RV-B	1	4	7,215	1.25
	ToMV	1	3	6,384	1.25
	TTMV	1	3	2912	3.43
	TTMDV	1	3	3256	2.15
	TTV	1	5	3725	2.01
	RV-C	5	15	7107	6.26
P176, P186, P213	WSSV	6	31	292,967	0.13
	SAFV	5	8023	8,115	90.92
	RV-C	8	37	7107	9.01
C46, P54	RV	3	12	17360	1.09
	TTMV	6	73	2912	15.45
	TTMDV	1	11	3256	2.15
	TTV	13	150	3725	50.74
	RV-C	3	9	7107	2.25
P19, P88	HHV	3	12	116114	0.18
	HPV	0	0	7466	0.00
	ToMV	1	7	6,384	1.25
	TTMDV	1	6	3256	3.38
	RV-C	10	8726	7107	62.77
T36	RV-C	5	21	7107	4.43
T33, T39	RV-C	12	1319	7107	68.74
T43, T44	RV-C	3	14165	7107	92.92
C06, C55, T78, V24	HASTV	2	5	6,759	2.52
	CMV	1	6	3356	2.09
	SAFV	2	126	8,115	2.42
	RV-C	3	7989	7107	92.66
V26	HBoV	8	77	5299	15.30
	RV-C	7	143	7107	15.06
C29, M40, M41, V39	SAFV	8	135	8,115	8.76
	TTMV	1	5	2912	2.75
	RV-C	11	3061	7107	63.89
P131, V61	BatPV	1	2	7,753	0.90
	HBoV	1	4	5299	2.08
	RV-A	3	14	7,129	3.23
	RV-B	4	3145	7,215	90.33
	HEV A	3	13	7,413	3.10
	TTMV	9	1531	2912	47.12
	TTMDV	4	1881	3256	29.67
	TTV	13	126	3725	42.31
	RV-C	4	618	7107	19.77

a RSV, respiratory syncytial virus; RV-A, rhinovirus species A; RV-B; rhinovirus species B; RV-C; rhinovirus species C; HBoV, human bocavirus, HCoV, human coronavirus OC43; TTV, torque teno virus; TTMV, torque teno mini virus; TTMDV, torque teno midi virus; BKV, bovine kobuvirus; BVDV, bovine viral diarrhea virus; BatPV, bat picornavirus; HPV, human papillomavirus; PVY, potato virus Y, ToMV, tomato mosaic virus; CMV, cucumber mosaic virus; HHV, human herpes virus; HVE-A, human enterovirus A; SAFV, Saffold virus; WSSV, white spot syndrome virus; HASTV, human astrovirus.

The assembled sequences from HCoV-OC43, RV, HBoV, Saffold, and anelloviruses were used to construct phylogenetic trees to determine the genetic similarity of the viruses characterized in this work with those in databases (see Materials and Methods). All viruses from LRTI and URTI grouped with clades formed by previously reported virus sequences (not shown). Anelloviruses could be readily classified as TTV, TTMV, or TTMDV, with some samples containing the three genera (Tables 4 and 5). All HCoV detected belong to species HCoV-OC43 and grouped with other known betacoronaviruses. HBoVs were all genotype 1, while the Saffold viruses detected in this work belonged to either genotype 2 or 3. Of interest, in the case of some RV, HBoV, Saffold, and anelloviruses, different contigs mapped to different clades, suggesting that recombination events are common in this type of viruses, as has been reported for RV [25].

## Discussion

Improvements in diagnostic methods have increased the rate of identification of viral pathogens in different clinical conditions, such as gastrointestinal, respiratory, or neurologic infections. However, despite these advances there are still a significant number of cases (20–50%), in which the etiologic agents are believed to be viruses, but the agent is not identified [26]. Previously, we reported the presence of a respiratory virus in about 71% of nasal samples obtained from children with LRTI and URTI (Aponte et al., manuscript in preparation; see also the Pathogen Detection section above), using a PCR method able to detect 15 different respiratory viruses. After PCR screening the virus-negative samples for the presence of respiratory bacteria, 89.6% of children with LRTI and 91.1% with URTI had at least one potential pathogen identified. These percentages were raised to levels close to 80% for viruses in both patient populations after NGS analysis of the double-negative samples, and essentially 100% of the samples had either a common respiratory virus or bacteria identified (in only one of 526 URTI samples DNA reads from a potential pathogen was not identified). It is interesting that 6 of the 8 samples from both LRTI and URTI that were negative for viruses after NGS had less than one million valid reads (Tables 2 and 3). Since the number of sequence reads directly correlates with the amount of nucleic acids present in the original sample [21], the absence of virus detection in these samples could represent false-negative results; it is likely that with a larger amount of sample, or deeper sequencing, respiratory viruses could have also been detected.

The samples that resulted negative for viruses by PCR, and subsequently determined as PCR-positive for respiratory bacterial pathogens, were not characterized by NGS, but it is reasonable to assume that a high percentage of them could have also been positive for viruses by deep sequencing. It is difficult, however, to determine with confidence, which, if any, of the detected pathogens could be responsible for the clinical respiratory symptoms observed. The virus or bacteria detected by these methods could be present in the patient as an asymptomatic carrier state or as causal agents of asymptomatic infections. Studies comparing the presence of respiratory pathogens in nasal specimens from healthy children will help to resolve this issue. In addition, PCR and NGS are such sensitive techniques that the presence of small amounts of viral targets may not necessarily have clinical relevance. An additional limitation of this study is the limited number of samples analyzed. Exploring the possibility to define cutoff levels represents the next necessary step for diagnosing viral respiratory infections using molecular tests [27]. It is important to mention, however, that for several of the RV and HCoV detected in this study high genome sequence coverages were achieved. This observation indicates that a high number of DNA reads, and probably also of virus particles, were present in the samples. These viruses could have been undetected by PCR due to mismatches in the diagnostic primers used.

Of interest, the classes of viruses found by NGS in patients with LRTI and URTI were very similar, although their frequencies were different in the two study populations. RV was more frequently found in URTI (19 of 25 samples) vs. LRTI (9 of 25 samples), while coronavirus was more represented in LRTI (11/25) than in URTI (3/25). Only one of the 46 samples of children with URTI was positive for RSV, while in 5 of 25 samples from children with LRTI RSV was detected. Saffold viruses, members of the picornaviridae family and cardiovirus genus, were found only in children with URTI. Since their initial description in 2007, these viruses have been shown to circulate worldwide, occur early in life, and involve the respiratory and gastrointestinal tracts. The association of these viruses with clinical symptoms is under investigation and requires additional epidemiological studies to clarify their pathogenicity [28]. Anelloviruses (TTV, TTMV, TTMDV) were found in both LRTIs and URTIs. Members of this family ubiquitously infect humans and establish persistent infections, although causal disease associations are currently lacking [10]. It is interesting to note that common gastrointestinal viruses such as astrovirus and rotavirus were found in some of the samples. It is not surprising though, since rotaviruses have long being suspected to reach the gastrointestinal tract via mouth and nose. In fact, some rotavirus infections have been associated with respiratory symptoms [29]. Finally, we found low amounts of DNA reads corresponding to animal and plant viruses. The number of these types of viruses is larger than previously reported in respiratory samples [21], although plant viruses have been found more abundantly in human feces [30]. Both, plant and animal viruses are thought to be derived either from consumed food or acquired from the environment.

The search for new viruses using NGS technologies in mammalian, avian, and in particular, human samples, has contributed to the identification of new viruses in animal reservoirs and in different conditions of disease [31]. However, the important effort invested in mammal and avian virus detection has only resulted in the discovery of variants of virus species, sister species to known viruses, and rarely genera. These observations contrast with the recent efforts to discover arthropod viruses, which have yielded widely divergent taxa that sometimes have even defined novel families [32]. Altogether, these observations and the presence of DNA sequence reads from common respiratory viruses or bacteria in essentially 100% of the samples collected from children with LRTI and URTI, suggest there is limited potential for the discovery of so far undescribed, clinically relevant, viruses associated to pediatric respiratory disease at least in the type of populations studied and with the sampling and diagnostic methods employed.
